# Disappearing everyday materials: The displacement of medical resources following disaster in Fukushima, Japan^☆^

**DOI:** 10.1016/j.socscimed.2017.09.011

**Published:** 2017-10

**Authors:** Sudeepa Abeysinghe, Claire Leppold, Akihiko Ozaki, Mariko Morita, Masaharu Tsubokura

**Affiliations:** a Global Public Health Unit, Chrystal Macmillan Building, University of Edinburgh, EH8 9LD, UK; b Minamisoma Municipal General Hospital, Fukushima, Japan; c Department of Research, Minamisoma Municipal General Hospital, Fukushima 975-0033, Japan; d Department of Surgery, Minamisoma Municipal General Hospital, Fukushima 975-0033, Japan; e Department of Anaesthesiology, Minamisoma Municipal General Hospital, Fukushima 975-0033, Japan; f Department of Radiation Protection, Minamisoma Municipal General Hospital, Minamisoma, Fukushima 975-0033, Japan

**Keywords:** Japan, Fukushima, Disaster, Medical staff, Hospital, Qualitative

## Abstract

This study draws upon interviews of medical staff working in the city of Minamisoma, Japan, following the 2011 Triple Disaster. It investigates staff responses to the disruption of material resources as a consequence of the disaster and its management. The disruption of spaces, and the loss of oxygen supplies, food, and medications impacted upon staff experience and the ability of institutions to care for patients. This resulted in a restructuring of spaces and materials as workers made efforts to reconfigure and reestablish healthcare functions. This is one of the few qualitative studies which draws upon the experience and perspectives of health workers in understanding material disruption following disaster. This is particularly important since this case did not involve the breakdown of lifeline infrastructure, but rather, brought to attention the way everyday material objects shape social experience. In highlighting these effects, the paper makes the case for the social scientific investigation of the impact of disasters on healthcare, shedding light on an area of research currently dominated by disaster medicine.

## Highlights

• Examines staff experiences of post-disaster healthcare.
• Documents key re-organizations of spaces and objects following disaster.
• Notes the central role played by loss of hospital resources.
• Relationship between health workers and the everyday objects of their workplace.

## 1 Introduction

On 11 March 2011 at 2:46 p.m., a magnitude 9.0 earthquake hit the Tohoku region of north-east Japan. Now known as the Great East Japan earthquake, this event caused significant damage to inhabitants and dwellings. The off-shore epicentre precipitated a series of tsunami waves – travelling as far as 10 km in-land - along the East coast, resulting in nearly 20,000 recorded deaths (Nakahara and Ichikawa, 2013). A further consequence was damage to the Fukushima Daiichi Nuclear Power Plant, and the resultant release of radioactive materials. The combination of these three events – referred to as the 2011 Triple Disaster – has had a long-lasting impact upon the affected region.

**Fig. 1:**
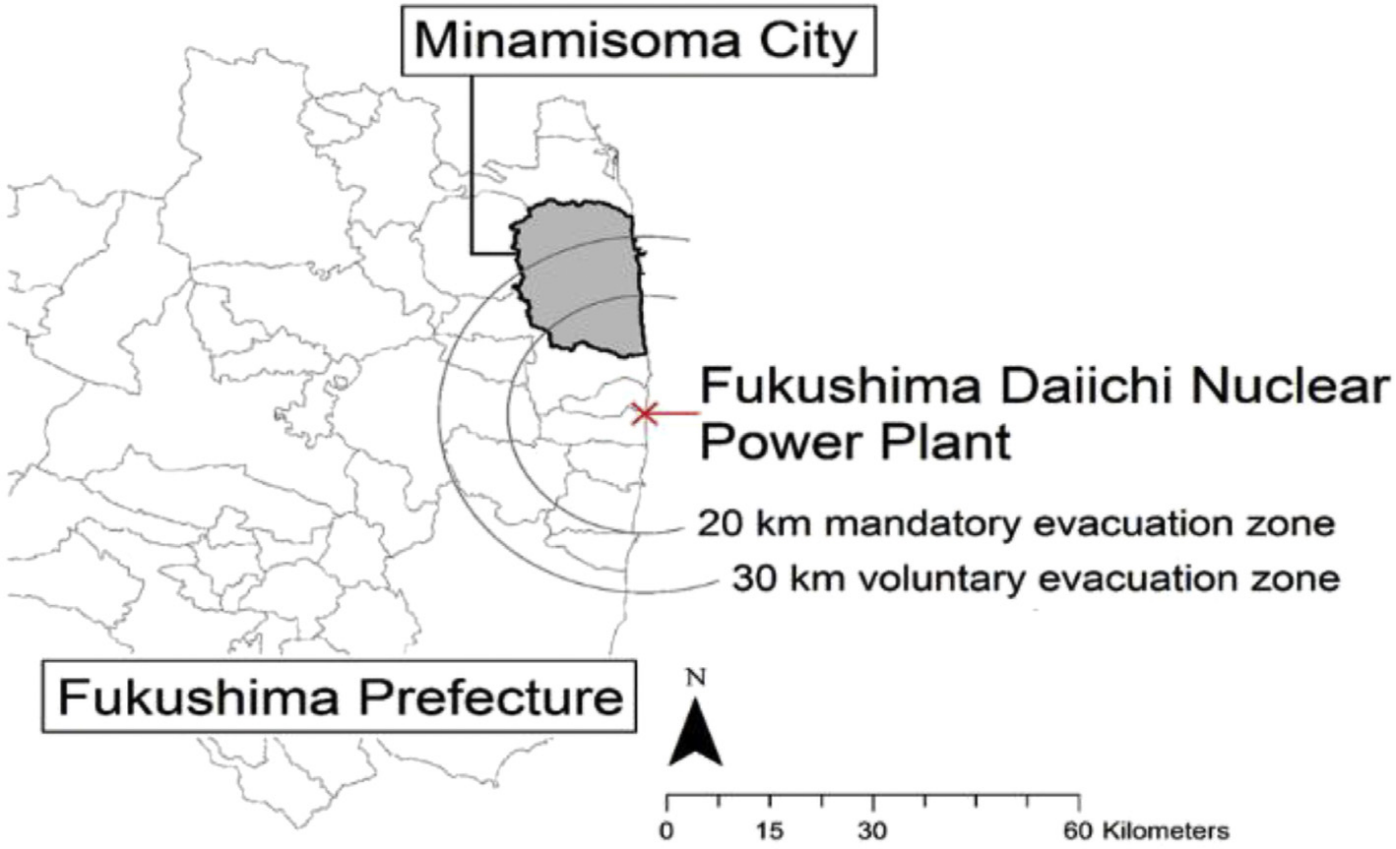
Evacuation Zones in relation to Minamisoma City.

This paper examines five health institutions across Minamisoma, a city with a ‘centre’ approximately 25 km from the Daiichi Plant, with (at the time of disaster) a relatively small population of approximately 71,000 (Zhang et al., 2014). The city spreads across three evacuation zones, created by the central government of Japan in response to the nuclear accident (Hasagawa, 2013). These were the 0-20 km Mandatory Evacuation Zone, the 20-30 km Voluntary Evacuation Zone (a liminal zone where evacuation was ‘voluntary’ but residents were subjected to indoor sheltering), and 30 + km non-evacuation areas (please refer to Fig. 1). Minamisoma is the closest regional centre to the Daiichi Plant and its placement across all three zones had an important impact on the experience of healthcare.

While there is a well-developed literature around the impact of disasters on healthcare institutions, this arises principally from the perspective of hospital management (Bar-Dayan et al., 2000, Wattanawaitunechai and Jitpratoom, 2005) and disaster preparedness (Kaji and Lewis, 2006, Manley et al., 2006). In particular, this literature emphasises the importance of maintaining ‘lifeline’ infrastructure – water, electricity, gas lines and buildings – in allowing hospitals to contribute to disaster response (Kai et al., 1994, Kuwata and Takada, 2003), and focuses on management decisions. Accounts of previous disasters, such as Hurricane Katrina (Klein and Nagel, 2007, Rodríguez and Aguirre, 2006) blackouts (Klein et al., 2005) and the Three Mile accident (Maxwell, 1982) point to the role of materialities, and their impact in creating and reinforcing inequalities. However, this literature too tends to focus on hospitals where lifeline infrastructure has been affected.

Also from the hospital management literature, work on the effects of disasters on healthcare staff empirically focus on quantitative investigation, for example accounting for health system breakdown, staff loss or in measuring stress (Qureshi et al., 2005, Wallace et al., 2007). There is little extant qualitative literature on the experience of staff in managing a disaster, with a limited literature of descriptive qualitative findings arising primarily from a nursing studies perspective (French et al., 2002, Sebastian et al., 2003). First person reports or narrative accounts also prevail. In respect to Fukushima, these have been produced by individual doctors (Nangaku and Akizawa, 2011) or hospitals (Irisawa, 2012, Koyama et al., 2011), and serve to highlight the role played by medical staff and provide insight from direct experience.

Social scientific analyses of disasters tend to focus on the social construction (Bankoff, 2004, Petryna, 2004, Stallings, 1995) or production (Perrow, 2011) of disaster risk, or the (inequitable) distribution of vulnerability (Barnshaw and Trainor, 2007, Bolin, 2007, Klinenberg, 2015; Phillips et al., 2010, Tierney, 2006, Wisner et al., 2004). A smaller literature examines the material experience of individuals who have survived natural disasters (Harada, 2000, Hastrup, 2010, Wilford, 2008), focusing on the way in which individuals rebuild social identities through (re)forming material surroundings and re-establishing home. There is an increasing and varied literature on the effects of the Fukushima disaster from sociological and anthropological perspectives (Gill et al., 2015, Hindmarsh, 2013), and within this an acknowledgement of the key role played by medical professionals. For example, Fortun and Morgan (2016), in making the case for comparative disaster studies, note the technical and operational challenges and communication issues faced by physicians post-Fukushima. Understanding the experiences of healthcare staff is key to fully accounting for the impact of disaster, and is a perspective that is currently under-examined.

While disaster management literature acknowledges the difficulties faced by medical professionals, this is under-studied through a use of qualitative data investigating the perspective of the actors themselves. This paper provides a qualitative sociological investigation of the experiences of these actors, which is novel in both the fields of disaster management and in medical sociology (where studies of disaster contexts are rare), despite calls for deeper sociological engagement with the problem of disasters (Tierney, 2007, Williams, 2008).

## 2 Methods

When the disaster occurred, Minamisoma was served by 8 hospitals and over 30 out-patient clinics. In combination, these institutions catered to the health needs of residents of the city and the wider rural area. Japanese universal health coverage operates under a pluralistic system; but, despite multiple sources of insurance, the national fee schedule means that payment is invariable regardless of where the care takes place (Ikegami et al., 2011, Ikegami and Campbell, 1995). Ikegami and Campbell (1995: 1296) note that “[v]irtually all physicians are in solo practice, and most hospitals are small, family enterprises that developed from physicians' offices. … [L]arge hospitals are owned by the national or local governments, voluntary organizations, and universities. For-profit investor-owned hospitals are prohibited.”

Two hospitals in Minamisoma provided to a large number and variety of patients. The first is a comprehensive public hospital which is situated in the 20-30 km ‘Voluntary Zone’. The second is a large private hospital which tended to deal with emergency and surgical cases as well as general medicine, also situated in the ‘Voluntary Zone’ at the time of disaster. Both the public and private hospital were of a similar size (between 180 and 240 beds). This paper documents the disaster's impact on these hospitals. It also rests on accounts from staff from three small private clinics, two of which have re-started operations, and one which is no longer in operation. The study is based on interviews with 35 medical staff who had been working at the point of the Triple Disaster, including doctors, nurses, allied health professionals, medical technicians, and administrative/support staff. The study focus is also informed by the secondary analysis of five informational interviews with medication suppliers and pharmacy operators conducted by Tsubokura.

One of the effects of the disaster – and of the confusion around radiation – was that a large proportion of medical staff evacuated the area (Kodama et al., 2014, Ochi et al., 2016). The region continues to suffer from a shortage of medical staff, one factor which makes understanding the disaster from the view of staff vital. At various points following the disaster fewer than 10 doctors remained in the public hospital, the largest research site. This limited the number of individuals available to interview. As such, and considering the high degree of saturation in the data around the issue of resources, this provides a representative account of staff experiences during the disaster. All remaining staff who were willing to be interviewed were actively recruited. The interviewees were enlisted by Ozaki, through a combination of formal recruitment (emails and personal communications), and by a snowballing from an initial sample and through gate-keepers. Ethics approval was granted by the committee of Minamisoma General Municipal Hospital (case number 28-6) and the University of Edinburgh. The research participants were provided with written (Japanese) information sheets. These were also verbally reviewed at the start of each interview, including the explicit consent for the interviews to be recorded and information on the extent of anonymization. Both verbal and written consent was obtained from each participant.

Each interview was conducted in Japanese with interpretation being provided between the English-speaking primary interviewer (Abeysinghe) and the interviewees. Interpretation was provided by Leppold, Ozaki and Morita with at least two interpreters being present at each interview to provide real-time checking. Transcripts were also checked to pick up additional translation issues prior to coding of interview data. All interviews were conducted by Abeysinghe, apart from one small-group interview of four medical technicians, who agreed to the interviewed only on the condition that they do so as a group. This interview was conducted in Japanese by Ozaki, with Leppold and Abeysinghe present, and subsequently translated. Translation can impact the nature of the data (e.g. choices about the most accurate translation of culturally-specific words/ideas and potential issues around implied meaning). However, the data was analysed using a broad thematic analysis, focusing on explicit narratives and key themes produced by the interviews, rather than discourse or semiotic analysis (for example), which would involve a close reading of language. All authors identified initial themes, which were coded and iteratively developed into sub-codes by Abeysinghe, following the approach to thematic analysis set out by Braun et al., (2012).

Interviews lengths ranged from 45 min to 2 h and 20 min following a semi-structured approach (Silverman, 2006) and were conducted in May-August 2016. The questions focused upon the interviewees' experience of their work during the time of the disaster. Questions generally prompted the interviewees to provide a semi-chronological account, allowing space to focus on moments which were critical to the interviewees' experiences of work and/or changes in job roles. Due to the disruption of the disaster, many staff changed positions or job status (e.g. moved hospitals or stopped working); occupation and location at the time of the disaster are cited here. This paper focuses upon data related to the period spanning 11 March to (roughly) 27 March 2011. This follows the impact of the earthquake and tsunami (11 March), nuclear accident (unfolding 12-15 March), evacuation of hospital in-patients (up to 20 March) and the situation immediately following evacuation.

## 3 Results

### 3.1 Initial disruption of materialities

Staff experiences focused around the key issue of material disruption. In understanding this aspect of staff experience, it was clear that the initial earthquake had a key impact in rearranging things and places. Recollections often dwelt upon the sudden disorder of materials. For nurses in both the clinics and the hospitals, some of the clearest memories from that period pointed to the physical reordering of their work environment:

Going to the wards, it was so surprising, because everything had been shaking so that the beds going in all directions, they weren't lined up. They were turned in odd directions. And so we went to check on all the patients, to make sure that no-one had fallen out or anything. But even fans from the ceiling had fallen down. (Nurse, Private Hospital)

As this example shows, the immediate disorder created by the earthquake created a point of initial concern.

The work following the earthquake therefore focused upon recreating order out of these things and spaces:

…there was actually a sixth-floor ward as well, but the sprinkler system had gone off, and there were all of these problems with water. So the other nurses and I, we were trying to … we had sheets and we were trying to deal with the water issues. And we were soaking up the water and squeezing out the sheets. Doing things like that to get rid of the water. (Nurse, Private Hospital)

Nurses in the clinics served similar roles immediately following the disaster:

Things had fallen down, so we just started cleaning up the clinic. It took a lot of time to clean everything. I'm not sure how many hours. Probably we just continued cleaning until the evening. I was cleaning the building and I kept going up to different floors and cleaning. (Nurse, Clinic)

This focus on restoring the material order of the workplace was dominant throughout nurses' accounts. In contrast, for doctors and technicians, immediate thoughts also turned to checking that medical technologies were still functioning:

So then, I … there was water coming out on the sixth floor, so I went to check on that. And I also went to check on the ventilation machines, and when I was doing that, the message came that a tsunami was about to come. (Doctor, Public Hospital)

So we were first checking on all of the machines, to make sure whether they could be used or not. … When we were looking at ours, it seemed like basically the CT scan and the MRI scan were essentially fine. (Technician, Public Hospital)

In the two hospitals, these more complex pieces of medical equipment – often among the first things that came to mind for staff after the shaking stopped - remained functional. Medical resources did not become highlighted as a point of concern until later in events.

The next key event was the growing understanding among staff that the disaster (at this point, now both the earthquake and tsunami) would produce an influx of new patients with injuries. This led to a purposeful rearrangement of resources and spaces. In preparing for these patients, staff re-conceptualised spaces and rearranged objects:

…the staff in our hospital moved all of the bed and medicines into the ICU. So even in the lobby of the out-patient office, we lined up all of these chairs and we made make-shift beds out of things. But after that, absolutely no patients came. Mmm … I thought, maybe it was like … you either die or you lived, and there is not really anything in between that. (Pharmacist, Private Hospital)

While the patients did not immediately appear, as had been expected, casualties did arrive the following morning. This led to alternative uses of space and of medical devices:

So we were contacted and told to bring things down to the ward to keep these people warm. Such as bags full of hot water. We were told to bring these to the first floor. And we were also told to bring the warming fluids or other equipment from the operating rooms. From the operating rooms, we bought these down to the first floor as well. (Nurse, Private Hospital)

The repurposing of space also altered the way in which care was provided:

I was going to do x-ray scans, and I remember walking the halls. And the halls were full of patients. There were patients everywhere. Even the operating theatres, where surgeries were not happening. Patients were in there too. On the floors. Usually you would put the patients on a bed and take an x-ray there. But we were taking x-rays with the patients just lying on the floor. (Technician, Public Hospital)

The social and professional outcome of the disaster was a remaking of space, technology and practices. This triggered the longer-term destabilisation of the places and objects of healthcare.

### 3.2 Missing materials

The initial disruption and disorder caused by the earthquake and tsunami were just a starting point to accounts of the disaster. For workers in the three clinics, these events led to the closure of their workplaces, and (following the radiation accident) their own evacuation out of their homes. For hospital workers, the closure of clinics led to a re-centring of the hospital as one of the few remaining sites of community healthcare. The nuclear power plant accident on the afternoon of March 12th placed additional burdens on the structure and function of the hospital.

Previously steady, stable and taken-for-granted resources came to the forefront of staff experiences. This period – where supplies of everyday hospital resources became destabilised – received the most emphasis in interviewees' accounts of the crisis of care. For example, one manager (following our explanation of the research) spontaneously opened his interview:

In terms of our hospital, the main problem was oxygen. People had to send us supplementary packages of oxygen, twice a week or so. The tanks we had were too small. So I think that was probably my biggest concern at the time of the disaster. We had a 5 tonne tank of oxygen and we have now added another 3 tonne tank, after the disaster. In total, there are now 8 tonnes. That was an important change. (Doctor/Manager, Public Hospital)

This example serves to highlight the central place of seemingly everyday hospital resources in managing the aftermath of the Triple Disaster. Issues with oxygen were a major point of concern across the hospitals in the city:

But even more than the medicines what was really tough was when the oxygen wouldn't come in.…Normally we had these big oxygen tanks in this area and we would use those. But we became unable to use those [as they started running low], and people were instead sending these really small oxygen tanks to us. At one point there were even efforts to use this device to distil oxygen from the atmosphere … (Pharmacist, Public Hospital)

For the hospital administration, disruption in supplies of oxygen became a major source of anxiety in maintaining the function of the hospital.

This shortage, rather than issues with medical technologies or personnel, became fundamental to the closure of medical sites. In accounting for the closure of the Private Hospital:

I think maybe if we had received some information, and some supplies, it would have been better to not close the hospital. The important factor was oxygen. We were getting this twice a week [normally]. That's the same for the oil for the heaters. Because of that, that's why I thought to close the hospital. [I: You weren't getting any of those supplies at all?] No. They didn't come at all. If they had come, I would not have left. If [listing the levels of government] had been able to supply those sorts of things, I think it would have been better for the lives of the patients to keep the hospital going. (Doctor/Manager, Private Hospital)

Lack of supply of basic resources was therefore fundamental to the crisis of care experienced within the city in the fortnight following the disaster. This disruption was a direct result of the enactment of the 30 km ‘Voluntary Zone’ beyond which many private supply companies refused to send vehicles and drivers. This led to a situation where there was a sizable population to be cared for in this area (since it was not an official Mandatory Evacuation Zone) but everyday supplies were diminishing. This posed an issue for remaining citizens (who were free to leave) but also placed a heavy burden on healthcare (where remaining staff felt it was their duty to stay, but where provision of care became increasingly untenable).

For staff working closely with patients, such as nurses and pharmacists, issues with food and fluids (in addition to oxygen supply, which tended to be focused upon by doctor-managers) were also critical to their work:

Even food for the patients would be coming in less and less. First one dish would be removed, and from the next meal another dish would be removed. Little by little the patients' food also decreased [in volume]. And so we were trying to use as little as possible. And when something ran out, we were just trying to use what we had to take care of the patients. For example, usually we used two or three bottles of fluid per day for the patients. But at this time they would get one bottle per day. (Nurse, Private Hospital)

The problem of food was fundamental to structuring the work experiences of some staff. The nurse-manager in charge of this effort commented:

The problem was how to prepare for food. Because we weren't making any food at the hospital [during normal operations], it was all delivered. And [during the disaster] the staff from the company that was preparing our food, they had all evacuated. So, I was then in charge of making kyushoku [‘school lunch’, here referring to hospital meals]. I was in charge of making three meals every day for around 200 people. But I had no experience in that … That was the most difficult thing. (Nurse/Manager, Public Hospital)

While doctors and technicians expressed most worry about the loss of medical supplies and oxygen, other categories of workers (in particular nurses) found the disruption in food supplies to be the principal factor in structuring their work. This restructuring in tasks across professional boundaries shows how the relationship between staff and their objects of work was also mediated by professional status.

What is significant is that in this disaster the infrastructure (buildings and lifeline supplies), technical materials (equipment) and staff were all still functioning within the hospitals. This therefore presents a contrast to cases where lifeline infrastructure has failed, as presented by much of the existing research on the breakdown of hospital function during disasters.

From the standpoint of medicine, as long as the doctors were there, we were able to medically take care of the patients. But there were some obvious things we couldn't do. Especially with the food, for diabetic patients or people who needed low-caloric meals, we couldn't control that at all. And for patients who had undergone surgery and needed liquid meals, we were completely unable do that either. (Nurse/Manager, Public Hospital)

This disruption influenced the ability to provide care, but also deeply impacted the lives of the staff themselves. For example, many staff members had been working, sleeping, eating and living in the hospital in the period following the disaster. This included staff whose homes had been rendered uninhabitable due to the tsunami or earthquake, and staff who had endured mandatory evacuation of their (often intact) homes due to the radiation crisis. For such staff, the breakdown of material supply also led to the loss of their use of the hospital as a temporary home:

…[I was living here] [f]rom when I first came here at the time of the disaster until the end of March. And at that point they told us that the staff couldn't stay here anymore, because it was too difficult to provide food. There wasn't enough food. So they were trying to reduce the amount of food they had to make. (Nurse, evac. to Public Hospital)

Loss of food and the resources of hospital life resulted in the continued reallocation of space in the days following the disaster. As well as affecting the hospital as a site of healthcare, this also has a significant impact of staff, particularly those who had taken up residence in the hospital itself. While the hospital had initially served as a refuge by those affected by the disaster, these functions deteriorated as the constraints imposed by the evacuation zones worsened.

### 3.3 Hospital evacuation and post-evacuation

The breakdown of supply which lead to the closure of the Private Hospital and other hospitals in the area resulted in the Public Hospital being designated a catchment site for remaining in-patients. Non-evacuated staff from the other hospitals began to work in the Public Hospital to care for their transferred patients. While this arrangement made sense in forming a single site for patient care and movement, it placed further strain on job roles and relationships and on the spaces and objects of the Public Hospital in maintaining care.

Since the initial destabilisation of space during the earthquake, the work areas of the hospital had not regained their original purpose. During the crisis, the movement of patients from several hospitals into the Public Hospital resulted in the repurposing of a ‘lecture theatre’ into a space for a large number of patients:

I think we probably had about 80 in-patients at that time. And when we got here [to the Public Hospital], probably about 40 of them – of the unconscious or bed-ridden ones – they were just put onto the floor. Futons were laid out for them, and those patients were just put onto the ground. These patients – yeah, there were around 40 of them – they were all in the … there is the big lecture auditorium room … Yeah, they were all laid out on the floor there. We didn't have beds for them. So we couldn't raise them up where they were lying down for feeding them or anything. So we had to pick the patients up ourselves and feed them ourselves. (Nurse, evac. to Public Hospital)

Associated with the re-purposing of spaces was the re-purposing of staff. Formal occupational roles were distorted and staff at all levels were undertaking work of other staff categories. In reflecting on the conditions of the ‘lecture room’ a clerical worker recalled:

For me, after we brought the patients here, they were actually in this room. The patients were actually laid out on chairs. I had never done this type of thing before, but I was even spoon-feeding the patients at that point. (Support Staff, evac. to Public Hospital)

The breakdown of objects and spaces at many of the hospitals in the city therefore led to further strain on the spaces of the Public Hospital and the staff (from all hospitals) who remained at the hospital to care for the patients.

Despite the considerable strain on staff and resources, the Public Hospital remained open to in-patients until the 19th March. At this point, the central government and hospital management agreed to the evacuation of in-patients from this hospital to beyond the 30 km zone. This order resulted in the evacuation of patients across two days (19 and 20 March). Given that the hospital had been under considerable stress during the post-tsunami period, it is perhaps unsurprising that this evacuation resulted in a breakdown of any remaining order and high levels of staff stress:

So then I was in charge of moving the patients around. And at one point we had a plan to move all of the patient to a different place. We had been told that the ambulances would come to evacuate the patients. So we moved all of the patients, to prepare them for these ambulances to pick them up. But the ambulances wouldn't show … they never showed up. So we had to take all the patients back to the wards. (Doctor/Manager, Public Hospital)

Ambulances had to be shared across the hospitals in the region. While the Public Hospital finally fully evacuated on the 19 and 20 March, a staff member from the Private Hospital recalls the shortage of ambulances to his workplace:

But on the last day, when we would call an ambulance and say ‘we need an ambulance now to take our patients’, they would not come. They said ‘oh, there are all being used by [Public] Hospital’. And they wouldn't come. And when I really think about it, [the Public] Hospital also received so many different things that we did not. Like the DMAT teams came, the military came, they were receiving things like oil for heaters. And that was completely different from [Private Hospital]. (Doctor, Private Hospital)

While ambulances and materials were scarce, this scarcity was not uniformly experienced. Here, the doctor notes that the DMAT teams established a base in the Public Hospital (thereby also providing that hospital with further supplies) while the other hospitals were not afforded similar public resources.

These disparities were also felt in relation to the evacuation of patients out of the disaster zone. In reflecting on this movement of patients, a manager of the private hospital reflects:

Our hospital's lifeline was complete. Electricity, gas, water, none of these things were damaged. So, if these things are complete, we didn't necessarily have to remove ourselves.

This was really difficult. And this becomes a little difficult to explain. But basically, the government didn't help us to move the patients at all. We just had to use our own connections; we had to make do. We brought buses from Shirikawa. Then we had ambulances to Izu. (Doctor/manager, Private Hospital)

The focusing of relief efforts on the central public hospital therefore had knock-on effects for the availability of other institutions in the city. In the end, this private hospital relied upon personal connections in moving their patients:

The reason the evacuation went well was because we were using the connections available to us. We asked people we knew. One thing is [local politician] - we talked in the middle of the night. And he said he would arrange ambulances and supplies.

The second thing is that the Director of [one specific] Hospital in Izu is one of my friends. I worked there before I worked here …. So we would always drink together. And he called me and asked, ‘are you alright’, and I said ‘well, we're not alright’. Then they said they would take the patients. (Doctor/manager, Private Hospital)

As such, there was a perceived disparity in the allocation of resources between the hospitals, and this was explained by the interviewees as an outcome of the focus of resources onto the public hospital and relative neglect of other institutions.

Again, disorder in staff roles is mirrored by disordered materialities. The breakdown of communications meant that ambulances did not arrive when expected (or at all), resulting in the movement and re-movement of patients across the wards of the hospital:

The things I was being told up here on the sixth floor were completely different to what the people down on the first floor would say. Sometimes we would take a patient down and they would say ‘oh, it's not this person. You have to take them back’. And so, we would bring them back the sixth floor. And then the people here would say ‘oh, we have to double-check’. And then they would call the first floor. And it would go like that. (Nurse, Public Hospital)

This again led to the re-ordering of materialities. The quote below, for examples, shows how buses were re-purposed as makeshift ambulances for unconscious patients:

There were a lot of patients who were unconscious or ones that were immobile. So for those patients, we just had the tourism buses. And we would have their stretchers and we would load them into the buses, where the stretchers would be across the top of the seat. So one stretcher over a pair of seat. I helped get these patients into the buses and I went with them on the bus. (Technician, Public Hospital)

Thus, one of the key impacts of the disaster on the hospital was the way in which spaces, things (and also work roles) fell into confusion. This had important implications on the way in which staff and patients experienced the crisis and in the abilities of healthcare institutions to maintain care in the city.

Following the evacuation of the hospital in-patients, there was still a sizable population remaining in the city. This of course meant that there were still health needs to be covered. The Public Hospital remained open for out-patient services to deal with those needs. To the surprise of many of the interviewed staff, out-patient clinics began to be primarily used by people seeking medications.

About 90% of people had been using outside pharmacies before the disaster. So we weren't used to giving medication to so many out-patients at the out-patient clinic at once. … There weren't that many types of medications in the hospital to start with. So there were lots of medications that had been used by other [specialist] hospitals … we didn't have those medications here for our patients. So for those cases, we were also able to find something else that was suitable. The doctors had given us permission to not even double-check with them. The doctors left it to us to find a suitable replacement for the scripts. (Pharmacist, Public Hospital)

This quote again shows the degree to which job roles became more fluid during the crisis. In this case, pharmacists (bypassing the need for doctors' orders) became involved in choosing medications due to the lack of some drugs and the (brand and active agent) substitution of other drugs.

As with the oxygen, oil and food, the disruption to the supply of medicine was a product of the establishment to the 30 km Voluntary Zone around the power plant. Some interviewees felt that medicines were simply not entering the area, though recollections on this aspect were varied:

Even if you asked for the medication, no-one would bring it here. But out of five times … maybe once or twice people would come. But then after the 30km radius – the indoor sheltering zone – well, the 30km radius became a zone that you could not enter. And from that order, even if people tried to bring things in, they couldn't. (Pharmacist, Public Hospital)

The decision to establish the evacuation zones made by the central government had a significant impact on the spaces, interactions and functions that could occur at the level of each health institution or each healthcare worker. While the loss of the supply of medication was felt and managed individually by the hospital or the staff, these small-scale actions were part of the larger picture of disruption to care in the region.

## 4 Discussion

The Triple Disaster fundamentally disrupted the material and spatial relations of health institutions. Through this disturbance, everyday work materials gained prominence in terms of staff experience. Resources and spaces became unstable, assumed alternate roles and altered in significance at different points in the crisis. The staff restructured and repurposed the materials, just as the loss of these materials restructured the realities and functions of the health institutions.

There is renewed medical social scientific interest in materialities (Fox, 1997, van Hout et al., 2015). This follows from classical literature on the symbolic role of objects in structuring professional boundaries (Becker, 1961) and examinations of the social history of medical artefacts (Pasveer, 1989). When examining the existing contemporary literature around the role of materialities within hospitals, these tend to focus upon novel, complex or contested medical technologies (Prout, 1996, Timmermans and Berg, 2003) despite explicit calls to examine the mundane within sociology (Latour, 1992) as well as to connect with other social scientific explanations, such as work on material culture (Miller, 2005). Sociologically, everyday healthcare resources such as food and fluid have been investigated in diverse (though few) studies (Feder-Alford, 2006, Harbers et al., 2002, McInerney, 1992, Thomson and Hassenkamp, 2008). On the whole, the roles played by hospital resources remain relatively unexamined within sociological accounts. This research serves to add to this literature around the important role played by material resources in forming the experience of healthcare work.

The study contributes to our understanding of the experiences of healthcare workers during times of crisis. Within the literature on disaster management, healthcare institutions are recognised as pivotal to a community's experience (Kaji and Lewis, 2006). In Minamisoma, it was clear that remaining healthcare institutions and personnel served a vital role in allowing citizens to remain in the ‘Voluntary Zone’ surrounding the nuclear accident site. Literature around the preparation for disaster highlights the importance of the lifeline infrastructure of the hospital (Kuwata and Takada, 2003). However, for both the hospitals focused upon in this study, these ‘lifeline’ materials were not disrupted. Yet the impact of the loss of food, and particularly oxygen for respirators, points to the need to more widely interpret the materials fundamental to a hospital's survival in disaster events. Just as important is to acknowledge the work performed by staff in restabilising these disrupted materials – transforming food, repurposing spaces and reorganising professional functions.

The loss of supply was not solely a result of the disaster itself but a consequence of the organisation of healthcare and of the response to the disaster. For example, staff from the private hospital reflected that they felt that private institutions were left to rely on personal contacts to try and negotiate resources, despite the fact that the operation of these hospitals is central to care in the region. According to these recollections, DMAT teams focused on the largest public hospital, leaving staff from the private hospital with the impression that they were not receiving similar assistance.

The analysis of the policy around the evacuation zones, and decisions to place resources at certain hospital sites, is beyond the scope of this paper. However, these aspects present an important area for future investigation in understanding the causes underpinning these experiences. This study shows how the bureaucratic ordering of place, through the central government's decision to draw up evacuation around the power plant, impacted deeply on the daily experiences of staff and patients during the crisis. The lack of supplies was directly related to this policy, as many suppliers were unwilling or unable to enter the Voluntary Zone. Ultimately, this resulted in the dangerous evacuation of patients (Nomura et al., 2015), disruption of work and personal lives, and destabilisation in the provision of healthcare. The situation, as captured in this research, was cause for high levels of staff stress, as individuals attempted to maintain healthcare functions in the face of the loss of supplies and in some case simultaneously managing the loss of their own homes and communities (through forced evacuation, as much as through the physical effects of disaster).

This research highlights the relationship between these workers and objects of their work, emphasising (through the disruption caused by the disaster) the often taken for granted networks of subjects and objects in stabilising the everyday practice of healthcare. A social scientific understanding of lived realities of the loss of seemingly mundane resources of hospital life adds an important perspective within the sociology of disasters and is indicative of the usefulness of health sociology (which currently has a limited interest in interrogating disasters) in investigating such contexts.

## 5 Conclusion

This study examined the experiences of hospital and clinic staff following the Fukushima Triple Disaster. In a context where lifelines infrastructures were functional, the loss of mundane materials had a profound effect of staff experiences of the disaster. In the case of the Private Hospital, issues with the supply of these goods was pivotal to the decision to close the facility. These issues are placed in a broader context of the official designation of evacuation zones, differences in the experiences of public and private hospitals and the culmination of individual-level decisions (about evacuation and about administering care during the crisis).

The experiences of Minamisoma medical workers highlights that the disruption caused by the disaster served in effect to emphasise previously taken for granted resources. The study points towards the need to further understand the role of these mundane resources and to examine the key part they play in the function of a healthcare institution and that they can play in time of crisis. It also highlights the need to further examine the experiences of health workers during conditions of crisis, in order to develop a deeper understanding of the experience of disaster, moving beyond hospital management perspectives towards a health sociological understanding.
